# A Novel Multistep Wavelet Convolutional Transfer Diagnostic Framework for Cross-Machine Bearing Fault Diagnosis

**DOI:** 10.3390/s25103141

**Published:** 2025-05-15

**Authors:** Lujia Zhao, Yuling He, Hai Zheng, Derui Dai

**Affiliations:** 1 Engineering Training and Innovation and Entrepreneurship Education Center, North China Electric Power University, Baoding 071003, China; 40852095@ncepu.edu.cn; 2 Department of Mechanical Engineering, North China Electric Power University, Baoding 071003, China; 220232224104@ncepu.edu.cn (H.Z.); 120232102035@ncepu.edu.cn (D.D.)

**Keywords:** bearing, fault diagnosis, cross-machine, transfer learning, wavelet convolutional network

## Abstract

Transfer learning has emerged as a potent technique for diagnosing bearing faults in environments with fluctuating operational parameters. Nevertheless, the majority of current transfer-learning-based fault diagnosis approaches focus primarily on adapting to varying conditions within the same machine. In real-world applications, there is a frequent need to extend these diagnostic techniques to machines that differ significantly in both function and structural design. Due to the different mechanical structures of different machines, the signal transmission paths are vastly different, and the distribution of collected data varies greatly, making it difficult for existing transfer fault diagnosis methods to meet diagnostic needs. Therefore, a multistep wavelet convolutional transfer diagnostic framework (MSWCTD) is proposed to realize cross-machine bearing fault diagnosis. Firstly, a multistep time shift wavelet convolutional network (MTSWCN) based on the multiscale technique and wavelet transform is proposed to explore the diversity information regarding original vibration data and enhance the feature expression ability. Secondly, a confusion transfer method based on multi-view learning is designed to extract diagnosis knowledge that is transferable, which reduces the discrepancy between machines. Three bearing datasets are utilized to evaluate the MSWCTD, with the MSWCTD showing excellent performance on cross-machine bearing fault diagnosis task.

## 1. Introduction

Within the domain of rotating machinery, including wind turbines, the reliability of bearings is crucial for stability. Failures may cause significant economic losses, and in severe cases, pose safety hazards. Despite their critical role, rolling bearings are inherently fragile components [1,2,3]. Thus, diagnosing faults in bearings is of utmost importance for ensuring the secure operation of devices. In recent years, intelligent fault diagnosis for rolling bearings has seen rapid progress with deep learning, achieving notable results. Most deep-learning-based diagnostic approaches for bearings are based on a basic assumption: that data distributions are similar [4,5]. However, in industrial applications, machines experience temporal variations in operational conditions, leading to data from various operational contexts. As a result, the data distribution is not uniform, and a model trained on one dataset may not consistently perform optimally when applied to other datasets.

Transfer learning is capable of effectively reducing distribution discrepancy and improving adaptability to data under different conditions [6]. In bearing fault diagnosis, transfer learning has been widely studied due to domain-shift problems caused by different operation conditions [7]. Xie et al. [8] combined Transfer Component Analysis (TCA) and SVM to achieve migration fault diagnosis. Zhang et al. [9] constructed a convolutional diagnosis model and completed fault migration diagnosis. Jiao et al. [10] comprehensively considered edge and conditional distribution and proposed a residual joint domain adaptive adversarial network. Wang et al. [11] adapted the probability distribution by reducing distance and completed cross-domain fault diagnosis tasks. Li et al. [12] used a convolutional neural network (CNN) [13] as the structure and the maximum mean discrepancy as the measurement method, which was able to achieve efficient bearing transfer fault diagnosis on the test dataset.

While the aforementioned transfer learning techniques have accomplished transferable fault diagnosis under different conditions, they have utilized data from a single machine. In the real world, however, data come from a variety of machine types. The discrepancies between datasets are not solely attributable to shifts in operating parameters but are also significantly impacted by intrinsic machine attributes and additional factors [14]. Therefore, the distribution difference leads to a decrease in transfer learning performance. There have been studies proposing new methods for cross-machine bearing fault diagnosis. Yang et al. [15] designed a transfer network using multi-layer maximum mean difference (MMD) to measure the difference in probability distribution, improving transfer diagnostic ability. Guo et al. [16] proposed a novel loss built on domain errors with probability designed for training the fault diagnosis method. Although the above methods have to some extent completed the diagnosis of transfer faults in rolling bearings between different machines, due to the large differences between machines, relying solely on transfer learning ideas to mine transferable knowledge still makes it difficult to improve accuracy. In addition, the above methods often consider reducing distribution differences from a single perspective of probability distribution, ignoring the multiple attributes of data. Therefore, we introduce mutual information to reduce data differences between different machines from multiple perspectives.

While the aforementioned methods can achieve bearing fault diagnosis across different machines, the models are trained solely based on data. These methods struggle to characterize the relationship between vibration signals and fault features. The lack of physical constraints in model training means that the features extracted for cross-machine fault diagnosis may not necessarily reflect the essence of the fault [17,18]. Wavelet transform, with its remarkable time-frequency analysis capabilities, has shown excellent performance in processing vibration signals and diagnosing bearing faults. However, the signal processing ability of wavelet transform is influenced by the scale factor and translation factor, and the selection of parameters is crucial for ensuring the accuracy of time-frequency feature description [19]. Although wavelet transform can provide accurate bearing fault diagnosis results when analyzing vibration signals, it relies heavily on expert experience. For example, the choice of wavelet basis function, determination of scale, and analysis of fault features all require a certain level of practical application experience. In recent years, integrating wavelet transform into neural networks has become a means for researchers to address the limitations of pure wavelet transform in signal processing. Fu et al. [20] designed the wavelet scattering transform layer to replace the conventional convolutional layer. The wavelet scattering transform layer utilized predefined Morlet wavelets to learn translation invariance features. Wang et al. [21] combined the wavelet kernel network with Bidirectional Long Short-Term Memory to utilize the respective advantages of both in mining significant characteristics related to bearing health status. He et al. [22] utilized wavelet technology to design the weight initialization of CNN. The parameters of wavelet transform were constrained and optimized by convolution operation. However, the wavelet-transform-based fault diagnosis method combined with neural networks mentioned above employs a single-scale wavelet basis. Since the vibration signals of mechanical equipment often contain fault information hidden across multiple scales due to the coupling of different structural components during operation, using a single-scale wavelet neural network may not be sufficient for extracting fault features.

In summary, combining transfer learning and a wavelet convolutional network may improve the accuracy under a cross-machine scenario. Therefore, the multistep wavelet convolutional transfer diagnostic framework (MSWCTD) consisting of the multistep time shift wavelet convolutional network (MTSWCN) and the multi-view confusion transfer method (MVCT) is proposed. The main contributions of this study are as follows:

1. A cutting-edge framework—MSWCTD is introduced for cross-machine fault diagnosis scenarios, which integrates a wavelet convolutional network and transfer learning techniques. This method is designed to handle two tasks, data reconstruction and fault diagnosis, to distill generalizable and transferable features for fault diagnosis across different machines. The performance of this approach is assessed using four distinct datasets.
2. A multistep time shift wavelet convolutional network (MTSWCN) based on wavelet transform and the time shift technique is proposed to explore the diversity of original vibration data and enhance feature expression ability. The proposed multistep time shift technique can fully utilize features extracted by MTSWCN and extract valuable features through the wavelet convolutional network. Furthermore, the multistep time shift technique improves data utilization and enhances diversity in feature extraction.
3. A multi-view confusion transfer method (MVCT) is proposed to obtain transferable knowledge of fault diagnosis and identify the health status of rolling bearings across machines. The method mines features from the perspectives of probability distribution and information to improve transfer diagnosis ability.

The remainder of this manuscript is organized as follows. Research related to the use of basic methods in this article is presented briefly in Section 2. MSWCTD is shown in Section 3. Three cases consisting of four bearing datasets are presented to affirm the efficacy of MSWCTD in Section 4. In Section 5, conclusions are drawn via the experimental results.

## 2. Related Works

### 2.1. Transfer Learning

The central goal of transfer learning is to capture reusable insights from a source domain and implement them in a target domain. Depending on alignment between the feature space and label space of different domains, transfer learning can be classified into homogeneous transfer learning, where they are consistent, and heterogeneous transfer learning, where they are not [23].

Currently, the majority of transfer-learning-based fault diagnosis methods across varying operating conditions focus on the probability distribution, aiming to align the data characteristics of different operating conditions by minimizing the disparities in their probability distributions. Zhang et al. [24] utilized multi-kernel MMD to minimize the distribution differences. Guo et al. [25] used the distribution measurement characteristics of MMD and applied it to evaluate generated signals. Fang et al. [26] adopted MMD and local MMD to align the data distribution and completed transfer fault diagnosis. Obviously, the above methods concerned the single measurement of data. However, relying solely on this single metric is insufficient to capture the full spectrum of data feature diversity. Consequently, we address the differences in data among various machines from a multifaceted perspective.

### 2.2. Wavelet Transform

The core idea of wavelet transform is to perform the multi-scale decomposition of signals through a wavelet basic function with adjustable scale and variable position, to analyze different frequency components and local features of the signal. With excellent signal processing capabilities, wavelet transform has been favored in fields such as image processing, speech processing, and vibration signal processing. Wavelet transform can simultaneously analyze the time-frequency characteristics of signals at multiple resolution levels, capture the transient features of signals, and effectively distinguish high-frequency noise and the low-frequency details of signals, and has been studied in bearing faults diagnosis [27].

Wavelet transform is mathematically defined as follows:

(1) $$W_{a,b}\left(t\right)=x\left(t\right)*\psi_{a,b}\left(t\right)$$

where $x\left(t\right)$ is the input signal, ∗ is the convolutional operation, t is time, a is the scale factor, b is the translation factor, and $\psi_{a,b\left(t\right)}$ is the wavelet function.

Different wavelet basis functions are suitable for analyzing different signal processing tasks, such as Morlet, Mexican Hat, Gaussian, Shannon and Laplace. In the analysis of bearing vibration signals, Morlet wavelet basis functions are the most commonly used [16]. The Morlet wavelet is defined as

(2) $$\psi_{a,b}\left(t\right)=\pi^{-\frac{1}{4}}e^{j2\pi f\frac{\left(t-b\right)}{a}}e^{-\frac{{\left(t-b\right)}^{2}}{2a^{2}}}$$

where $\pi^{-\frac{1}{4}}$ is the normalization coefficient, f is the central frequency, and $e\left(\cdot \right)$ is the exponential function. Substituting Equation (2) into (1), the continuous Morlet wavelet transform is defined as follows:

(3) $$W_{a,b}\left(t\right)=x\left(t\right)*\psi_{a,b}\left(t\right)=\pi^{-\frac{1}{4}}\int_{-\infty }^{+\infty }x\left(t\right)e^{j2\pi f\frac{\left(t-b\right)}{a}}e^{-\frac{{\left(t-b\right)}^{2}}{2a^{2}}}dt$$

## 3. The Multistep Wavelet Convolutional Transfer Diagnostic Method

**Figure 1:**
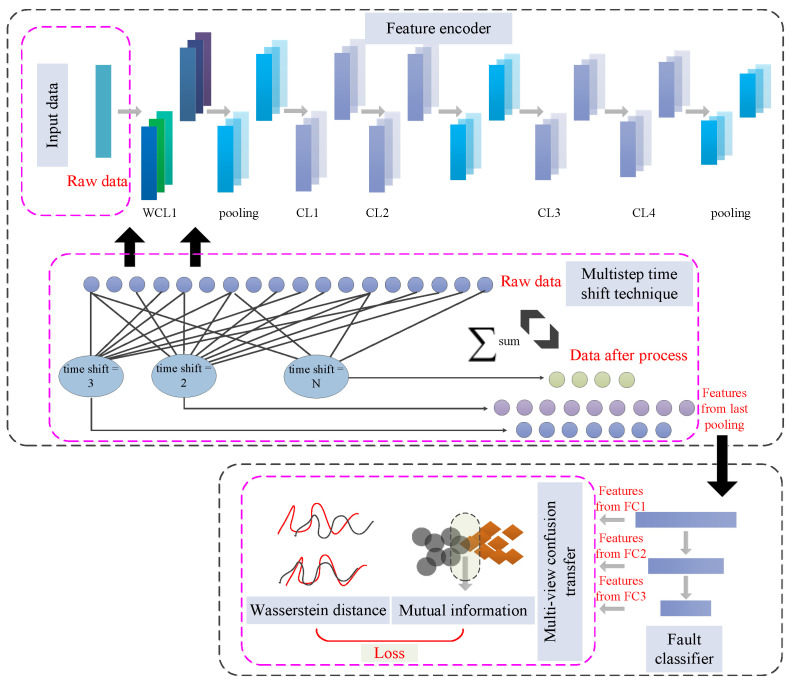
The framework of MSWCTD.

The multistep wavelet convolutional transfer diagnostic framework (MSWCTD) contains a feature encoder, a multistep time shift module, and a classifier, as shown in Figure 1. Among them, the basic framework of the feature encoder is the wavelet convolutional network. The proposed multistep time shift technique is utilized in the input layer and the first wavelet convolutional layer. The wavelet convolutional network [28] consists of one wavelet convolutional layer (WCL1) and four convolutional layers (CL1–CL4) [29]. The classifier consists of three full-connected layers (FC1–FC3). The parameters are presented in Table 1.

### 3.1. The Procedure of MSWCTD

**Figure 2:**
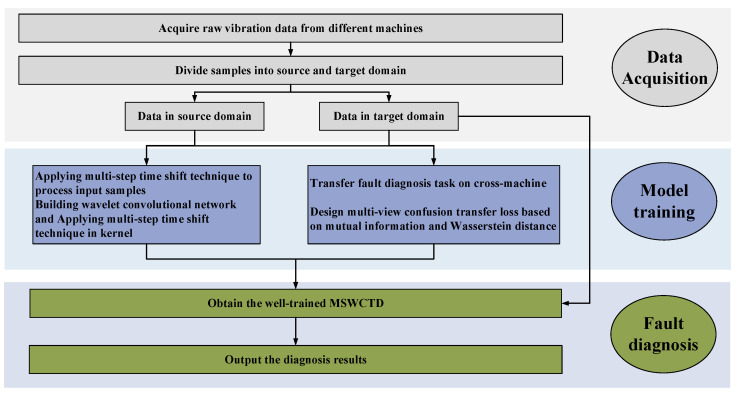
Fault diagnosis process of MSWCTD.

The MSWCTD methodology, as delineated in Figure 2, encompasses three principal phases: data acquisition, model training, and health identification. The overarching steps of MSWCTD are as follows:

**Step 1:** In data acquisition, a signal acquisition system is employed to gather a spectrum of vibration signals indicative of the health status of rolling bearings across different machineries, categorizing them into a source domain dataset for training purposes and a target domain dataset for evaluation.

**Step 2:** In model training, both source and target domain datasets are input into constructed fault diagnostic model simultaneously. The model utilizes multi-view confusion transfer loss based on WD and mutual information to optimize the parameters to give it good performance.

**Step 3:** In fault diagnosis, the well-trained model is used to identify types of health condition on the target domain dataset.

The Algorithm 1 of MSWCTD is listed as follows.

**Algorithm of the proposed method**:
**Algorithm 1:** Datasets from different machines1. Randomly initialize: parameters *θ* of the proposed method 2. while not reaching the maximum number of iterations do: 3. calculate the output in Equation (6) 4. calculate the loss of transfer learning in Equation (9) 5. calculate the final loss in Equation (10) 6. update parameters with gradient descent:
$\theta^{\prime }=\theta -\alpha \nabla L(\theta )$, where α is the learning rate. 7. end while

### 3.2. Multistep Time Shift Wavelet Convolutional Network

The multistep time shift wavelet convolutional network (MTSWCN) employs a multistep time shift technique to alter the size of wavelet convolutional kernels. Unlike existing multi-scale convolutional kernel operations, the proposed multistep time shift technique does not simply place wavelet convolutional kernels of different sizes on separate branches of the network structure. Instead, it divides the kernel size within the same layer of the wavelet convolutional network and segments it according to an equidistant ratio. And unlike existing wavelet neural networks that only use a determined single size kernel for feature extraction, MTSWCN uses multi-scale wavelet convolution kernels to extract multi-scale information. This allows the features extracted by wavelet convolutional kernels with different time shifts to be superimposed. To enhance data utilization, the input samples are also divided using the multistep time shift method and then randomly recombined. However, to ensure the integrity of information, each segmented sample must be superimposed with the original signal sample.

Therefore, in the input layer, given the sample $x=\{x_{1},x_{2},\ldots ,x_{n}\}$, *n* is the length of sample. The output of different time shift steps can be calculated as

(4) yτ={x1,xτ+1,x2τ+1,…,xNτ+1},N=nτ−1,τ=2k,k∈ℤ+

where τ is the time shift step and $y_{\tau }$ denotes the sample after dividing.

After undergoing the multistep time shift in the input layer, the samples are randomly recombined and superimposed with the original samples, defined as follows:

(5) $$y=\sum_{N=1}\mathrm{rand}(N)+x$$

where y is the output of the input layer and rand(*g*) denotes random number operation.

Applying the multistep time shift technique to the wavelet convolutional kernel, the output can be denoted as

(6) $$W_{a,b}(t)=\sum_{\tau =1}y(t)\times \psi_{a,b}(\tau )$$

## 3.3. Multi-View Confusion Transfer

Maximum mean discrepancy (MMD) is a prevalent technique for assessing probability distributions which is frequently employed in transfer learning to quantify the divergence in data distributions across various domains. The calculation of MMD is defined as follows:

(7) $$MMD(X,Y)=\frac{1}{n^{2}}\sum_{i=1}^{n}\sum_{j=1}^{n}k(x_{i},x_{j})+\frac{1}{m^{2}}\sum_{i=1}^{m}\sum_{j=1}^{m}k(y_{i},y_{j})-\frac{2}{mn}\sum_{i=1}^{n}\sum_{j=1}^{m}k(x_{i},y_{j})$$

where k is the characteristic kernel and ϕ(⋅) is the nonlinear transformation from original space to reproducing kernel Hilbert space. *n* is the number of samples in X, and M is the number of samples in Y.

This serves as an optimization target for training models, aiming to minimize the distributional discrepancies between domains. However, MMD’s reliance on feature mapping to a new space poses challenges for updating parameters of the operation. In contrast, the Wasserstein distance (WD) [30] does not have this limitation. The WD is a classical measure used to determine the shortest path, and it is crucial in comparing probability distributions. It is defined as

(8) $$WD\left(P(X),P(Y)\right)=\underset{\mu \in \prod \left(P(X),P(Y)\right)}{inf}E_{\left(x,y\right)\sim \mu }\left[\left\|x-y\right\|\right]$$

where $\prod \left(P(X),P(Y)\right)$ is the joint distribution μ(x,y) of variables $\left(x,y\right)$, P(X) and P(Y) are the marginal distribution, and $\inf\left(\cdot \right)$ is the infimum.

The larger the mutual information value, the greater correlation between random variables. Given variables *A* and *B*, the joint probability distribution is P(A,B), P(A) and P(B) are the marginal probability distribution of *A* and *B*, respectively, and the mutual information between *A* and *B* is defined as

(9) $$I(A;B)=\sum_{a\in A}\sum_{b\in B}p(a,b)\log\left(\frac{p(a,b)}{p(a)p(b)}\right)$$

Consequently, by diminishing the disparities from a probabilistic standpoint, the model can explore domain-adaptation features with similar probability distributions. This is exactly the principle followed by most domain adaptation transfer learning fault diagnosis methods. Unlike domain adaptation methods, we also approach this from an information perspective by integrating mutual information loss terms. By maximizing the extraction of common information from the perspective of information entropy, the model can acquire useful fault knowledge with a high degree of informational correlation. By amalgamating considerations of probability distribution and mutual information, multi-view confusion transfer (MVCT) is proposed. It becomes feasible to excavate transferable general knowledge from cross-machine bearing data, thereby enhancing the capacity of model. The criterion is calculated as

(10) $$L_{trans}=WD(P(S),P(T))-I(S;T)$$

where P(S) and P(T) represent the probability distribution of the source and target domain. I(S;T) denotes mutual information extracted by the model from cross-domain data.

## 3.4. The Loss Function of MSWCTD

MSWCTD encompasses both transfer and classification tasks as the loss basis. Two loss functions are utilized to train the model for cross-machine fault diagnosis. For transfer tasks, Equation (9) is chosen as the loss function to reduce the distribution difference between different domains. Conversely, the cross-entropy loss function is selected to diagnose fault modes. The cross-entropy can be calculated as

(11) $$L_{class}=-\frac{1}{M}\sum_{i}\sum_{k=1}^{N}y_{ik}\log(p_{ik})$$

where M is the number of samples, N is the number of fault types, and $y_{ik}=1$ if the true label of the ith sample is k, otherwise $y_{ik}=0$. p is the probability value of the model output.

The final loss of MSWCTD is denoted as

(12) $$L=L_{class}+L_{trans}$$

where L is the final loss of MSWCTD.

## 4. Case Verification

### 4.1. Case 1: CWRU and Ottawa

#### 4.1.1. Dataset Description

**Figure 3:**
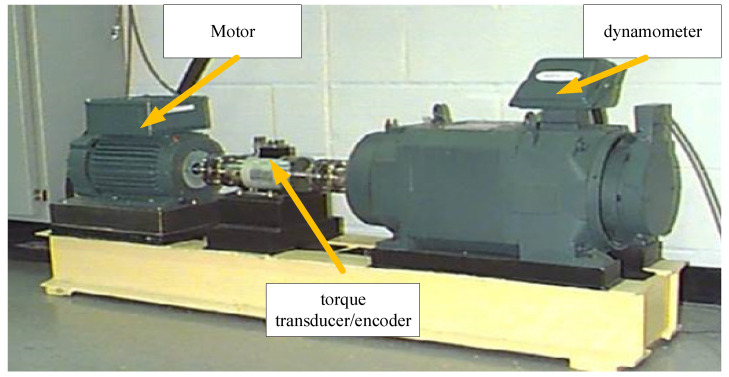
Experimental setup of CWRU.

Recently, the benchmark rotating machinery fault dataset, extensively utilized in the field, was procured from Case Western Reserve University (CWRU) [31]. As depicted in Figure 3, the experimental configuration was primarily composed of an electric motor, a torque transducer/encoder, and a dynamometer. Single-point defects in the rolling bearings were induced via the electro-discharge machining method. The employed data corresponded to a 0 hp motor operating at speed of 1797 rpm. Data collection was performed using an accelerometer transducer, which was mounted atop the bearing housing. This dataset includes three fault categories, which include normal (N), inner race (IF), and outer race (OF) faults. Each category contains 40 samples, with 1024 data points in each sample.

**Figure 4:**
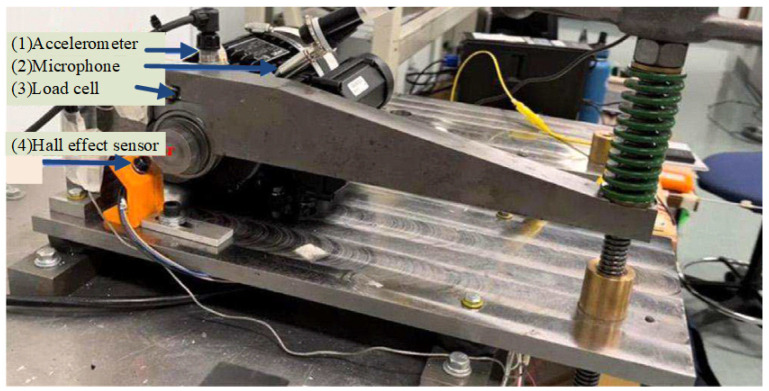
Experimental setup of Ottawa.

**Figure 5:**
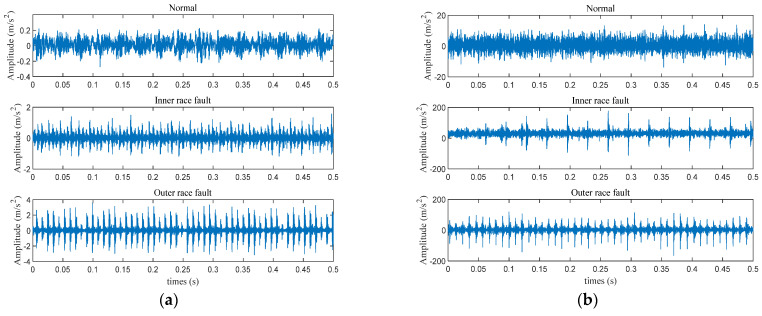
The time-domain waveform: (**a**) CWRU; (**b**) Ottawa.

Another dataset was from the University of Ottawa [32]. As depicted in Figure 4, it includes a single-phase motor attached to a solid plate, which is supported by vibration isolation mounts. The shaft extends through a coupling. The motor runs at a constant speed of 1750 rpm. Data collection was performed at a sampling rate of 42 kHz, with each condition lasting 10 s. The bearings in this experiment demonstrate three states of health: normal, inner race failure, and outer race failure. Each state is represented by 40 instances, each containing 1024 data points. Initial signal patterns under various conditions are shown in Figure 5b.

We designed a cross-machine fault diagnosis experiment, and the configuration is shown in Table 2.

#### 4.1.2. Result Analysis

In order to evaluate the MSWCTD, a comparative analysis is conducted with the Deep Adaptation Network (DAN), EWSNet [28], and DCC [33], as well as Transfer Component Analysis (TCA). The main reason for choosing these methods is that TCA and DAN are classic transfer learning methods with representativeness. DCC is a relatively cutting-edge transfer learning method with progressiveness. EWSNet is a cutting-edge wavelet neural network method, and the above methods can be compared to demonstrate the powerful transfer feature extraction ability of MSWCTD. The configurations and parameters of the networks are as follows:

1. TCA represents a seminal approach within the realm of transfer learning, employing MMD as its metric for aligning cross-domain data into a unified space to assess distributional disparities, without the integration of deep learning techniques.
2. The foundational architecture of the DAN aligns with that of the proposed technique, with the key distinction being the absence of a decoder component. It employs multi-kernel MMD and assesses disparities in the multi-layer output features.
3. EWSNet and DCC are the same as in the raw literature.

The comparative experimental outcomes among various methods are presented in Table 3. MSWCTD (our model) demonstrates superior accuracy in T-A and T-B. This suggests that the MSWCTD possesses robust transfer fault diagnosis capabilities. The main reason for this may be that the integration of an informatics-based loss function in the design facilitates better model training compared to singular probability distribution assessment methods. MSWCTD can bolster the ability to learn transferable knowledge. Comparing the experimental results obtained by EWSNet and DAN, although EWSNet did not use migration methods, its fault diagnosis accuracy was higher than DAN’s. This indicates that using some information processing techniques to integrate neural networks can improve the ability to extract generalized features, which can highlight the importance of fault information and thus extract essential features from data regarding similar fault conditions in different machines. From Table 3, the accuracy of TCA is the worst. The reason for this result is that TCA relies solely on solving MMD to reduce distribution differences, lacking adaptive capabilities.

**Figure 6:**
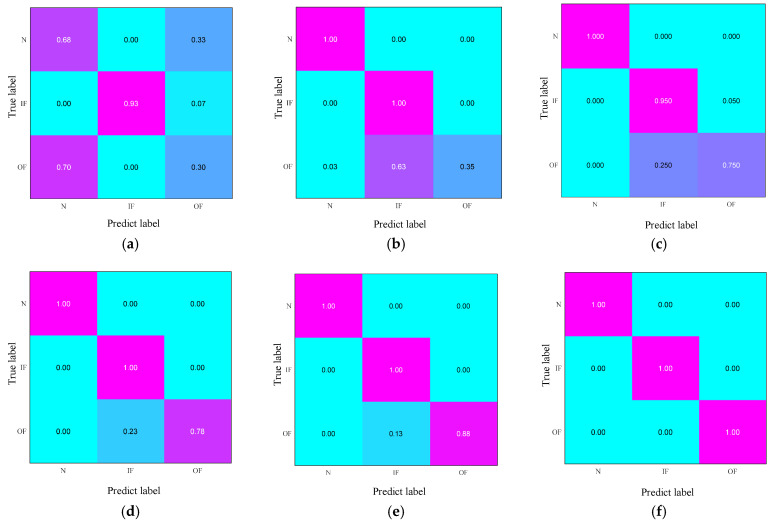
The confusion matrix: (**a**) TCA; (**b**) DAN; (**c**) EWSNet; (**d**) DCC; (**e**) Ref. [35]; (**f**) MSWCTD (this paper).

To assess the discriminatory capacity of the five methods across various health states, a confusion matrix was employed to illustrate their performance, as depicted in Figure 6. Figure 6f presents the recognition outcomes of MSWCTD. The diagonal figures indicate a recognition accuracy of 100% for the different health states. The findings suggest that (1) MSWCTD is adept at extracting knowledge that is transferable and can be easily adapted for use with data from novel machinery and (2) it is proficient at identifying features that are indicative of different health states, accurately pinpointing the health status. In contrast to other methods that can accurately detect outer race faults, the result of TCA is marginally lower, signifying the limited capability to learn distinctive transferable knowledge.

**Figure 7:**
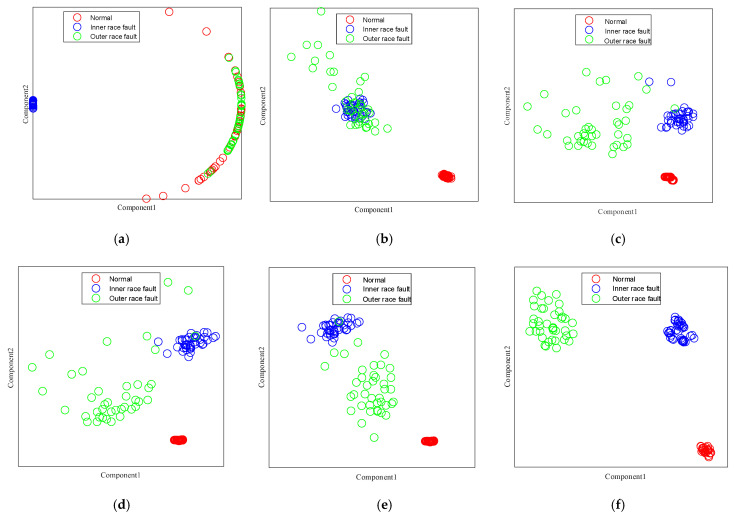
(**a**) TCA visualization; (**b**) DAN visualization; (**c**) EWSNet visualization; (**d**) DCC visualization; (**e**) Ref. [35] visualization; (**f**) MSWCTD (this paper) visualization.

To distinctly visualize the experimental results, t-distributed stochastic neighbor embedding (t-SNE) [34] was utilized to render the output, as illustrated in Figure 7. Optimally, performance is considered better when features of identical health states are closely aligned and those of disparate health states are more distant from one another. As shown in Figure 7a, only the inner ring fault is accurately distinguished. Upon comparing Figure 7a–f, the separability of different features in Figure 7f is the most pronounced, with a relatively large distance between different conditions, and the features of the same condition are tightly clustered, suggesting that MSWCTD can fully handle cross-machine diagnosis tasks. Some outer ring features are scattered among the inner ring fault features, as observed in Figure 7d. This scattering can lead to model misclassifications when differentiating between the two health states, indicating a lower fault diagnosis capability.

**Table 3:** Accuracy on two experimental tasks.

Approach	Accuracy on T-A (%)	Accuracy on T-B (%)
TCA	63.33	59.17
DAN	78.33	74.17
EWSNet	90.00	91.67
DCC	92.50	88.33
Ref. [35]	95.83	95.00
MSWCTD (this paper)	100.00	98.33

#### 4.1.3. Ablation Experiment

To highlight the superiority of the different innovative aspects of the proposed method and their contributions to the method’s performance, we designed ablation experiments. The methods used in the ablation experiments were as follows:

(1) WCN-MVCT: A fault diagnosis model built by combining a single-scale wavelet convolutional network (WCN) with the multiview cross-domain transfer (MVCT) method.
(2) MTSWCN-WD: A fault diagnosis model constructed by combining the multiscale time-shifted wavelet convolutional network (MTSWCN) with a single weighted distance (WD) metric.
(3) MSWCTD: The method proposed in this paper.

The results of the ablation experiments are shown in Table 4. From this table, it can be seen that the accuracy of MTSWCN-WD is on average higher than that of WCN-MVCT. This indicates that MTSWCN contributes more to improving the model’s transferable fault diagnosis performance compared to MVCT, and it also demonstrates the importance of feature extraction capabilities for cross-machine fault diagnosis models. Between MTSWCN-WD and the proposed method, MSWCTD achieves higher precision, which shows that MVCT can overcome the data bias caused by different mechanical structures and is more capable of focusing on sufficient transferable features than a single-metric approach. By comparing WCN-MVCT and MSWCTD, it can be observed that the WCN improved with the multistep time shift technique can extract transferable features related to faults and fully utilize multiscale information to enhance the model’s fault diagnosis capability.

### 4.2. Case 2: SEU and CWRU

#### 4.2.1. Dataset

The SEU data used in this study were obtained from the gearbox dataset procured at Southeast University [36]. This study extracted a subset of bearing data under two distinct operating conditions, as shown in Table 5. These conditions were defined by a speed–load configuration: one being 20 Hz–0 V (0 Nm) and the other 30 Hz–2 V (7.32 Nm). Sampling frequency was 5120 Hz. Three types of faults were chosen, i.e., normal operation (N), inner ring failure (IF), and outer ring failure (OF), with 40 samples for each condition and a sample size of 1024 data points per sample.

#### 4.2.2. Result Analysis

To substantiate the efficacy of MSWCTD, comparisons with TCA, DAN, and EWSNet were conducted, along with DCC. The network architectures and parameters employed in these experiments are consistent with those detailed previously.

**Figure 8:**
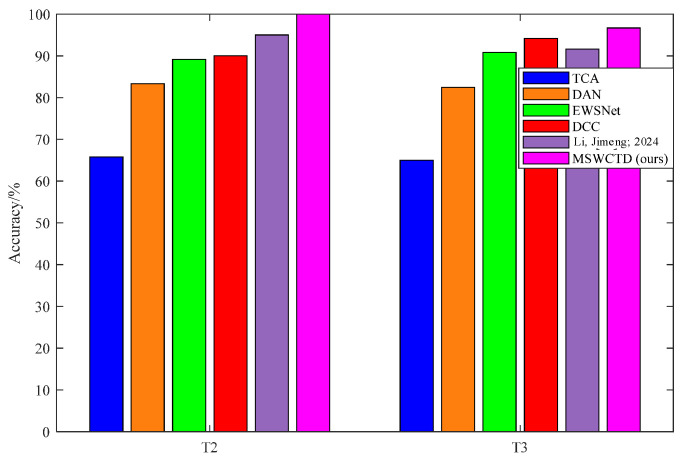
Results of contrastive methods. Li, Jimeng’s 2024 method is referenced [35].

Experimental outcomes are presented in Table 6 and Figure 8. The results indicate that MSWCTD achieves an accuracy of 100% in task T-C and 97.67% in task T-D, surpassing the recognition accuracy of others. It suggests that MSWCTD possesses a notably robust capability. It is evident that EWSNet ‘s accuracy is considerably higher than DAN’s. This superiority is attributed to the utilization of wavelet transform to extract powerful intrinsic features related to faults in the time-frequency domain with strong representation performance. As depicted in Figure 8, TCA exhibits the lowest diagnosis accuracy, primarily because TCA lacks adaptive learning capabilities and is unable to uncover deep-seated fault features.

**Figure 9:**
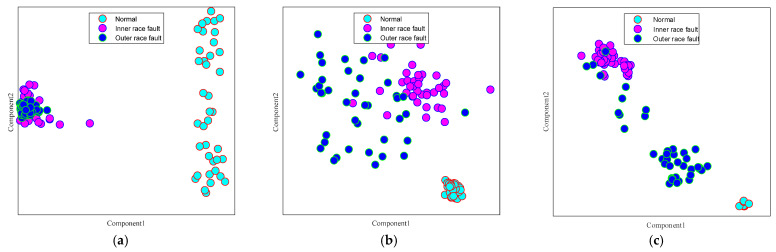

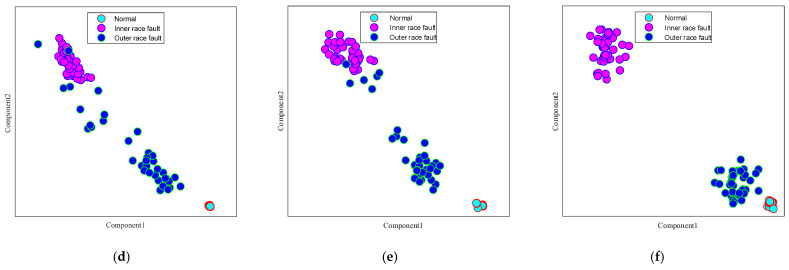
Visualization results: (**a**) TCA; (**b**) DAN; (**c**) EWSNet; (**d**) DCC; (**e**) Ref. [35]; (**f**) MSWCTD (this paper).

To distinctly visualize the cross-machine fault diagnosis performance for bearings, t-SNE visualization was applied, and the effect was as depicted in Figure 9. In Figure 9a, only normal health condition is accurately identified, while the inner and outer ring faults are challenging to differentiate. In Figure 9a–f, it is evident that Figure 9f exhibits the highest separability among the health conditions, with a comparatively large distance between different health states. The features corresponding to identical operating conditions are grouped together, suggesting that MSWCTD achieves superior classification performance and accurately discerns the various health conditions. Figure 9d indicates that, while the majority of inner and outer ring fault features are tightly concentrated, some outer ring features are dispersed among inner ring fault features. Dispersion increases the likelihood of misclassification when differentiating between the two health states, indicating a reduced cross-machine transfer diagnosis capability.

#### 4.2.3. Computational Efficiency

To further verify the computational efficiency advantages of MSWCTD, comparative experiments were designed considering model parameters, floating point operations (FLOPs), and inference time. The experimental results are shown in Table 7. As can be seen from the table, MSWCTD has an inference time of 0.56 s, which is lower compared to the two state-of-the-art methods, Ref. [35] and EWSNet. In terms of model parameters and FLOPs, MSWCTD does not significantly lag behind other methods, indicating that MSWCTD has certain advantages for industrial application.

## 5. Conclusions

A novel multistep wavelet convolutional transfer diagnostic framework (MSWCTD) is developed to realize the cross-machine fault identification of bearings. A multistep time shift wavelet convolutional network (MTSWCN) based on the multistep time shift technique and wavelet transform is proposed to explore the diversity of original vibration data and enhance the feature expression ability of models. The information relations of different network branches are considered to improve model complexity. In the fault diagnosis task, a multi-view confusion transfer method (MVCT) considering information and probability is designed to complete transfer diagnosis. By integrating the data probability distribution and information entropy in a comprehensive manner, the model demonstrates robust transfer learning capabilities and is able to fully exploit generalized transferable knowledge. MSWCTD is capable of not only thoroughly uncovering the fault-attribute characteristics embedded in vibration signals but also acquiring generalization transferability knowledge, thereby enhancing the accuracy of cross-machine rolling bearing fault diagnosis and achieving good results with an average accuracy exceeding that of the compared methods by 5%.

We did not explore the performance of our model under the influence of noise. In future work, we will further consider the influence of noise on diagnostic accuracy in practical applications and may use domain generalization techniques to improve the robustness of the model.

## Figures and Tables

**Table 1:** Detail parameters and structure of MSWCTD.

Modules	Description of Layer	Parameter
Feature encoder	Kernel shape of WCL1	1 ∗ 64
	Channels	(1,32)
	Kernel shape and stride of pooling operation	2 ∗ 2/2
	Kernel shape of CL1	1 ∗ 3
	Channels	(32,32)
	Kernel shape of CL2	1 ∗ 3
	Channels	(32,32)
	Kernel shape and stride of pooling operation	2 ∗ 2/2
	Kernel shape of CL3	1 ∗ 3
	Channels	(32,16)
	Kernel shape of CL4	1 ∗ 3
	Channels	(16,16)
	Kernel shape and stride of pooling operation	2 ∗ 2/2
Classifier	The neurons in FC1	16 ∗ 120/1024
	The neurons in FC2	1024/256
	The neurons in FC3	256/3

**Table 2:** Description of Case 1.

No.	Source Dataset	Target Dataset	Health Condition	The Number of Samples
T-A	CWRU	Ottawa	Normal	40
			Inner race fault	
			Outer race fault	
T-B	Ottawa	CWRU	Normal	40
			Inner race fault	
			Outer race fault	

**Table 4:** Accuracy of ablation experiment.

Approach	Accuracy on T-A (%)	Accuracy on T-B (%)
WCN-MVCT	86.67	85.83
MTSWCN-WD	90.83	91.67
MSWCTD (this paper)	100.00	98.33

**Table 5:** The detailed experimental setup using the different datasets.

No.	Source Dataset	Target Dataset	Health Condition	The Number of Samples
T-C	CWRU ^1^	SEU	N	40
			IF	
			OF	
T-D	SEU ^2^	CWRU	N	40
			IF	
			OF	

^1^ CWRU Case Western Reserve University Bearing data dataset; ^2^ Transmission data set obtained by Southeast University [36].

**Table 6:** Accuracy of different methods.

Method	Accuracy of T-C (%)	Accuracy of T-D (%)
TCA	65.83	65.00
DAN	83.33	82.50
EWSNet	89.17	90.83
DCC	90.00	94.17
Ref. [35]	95.00	91.67
MSWCTD (this paper)	100.00	96.67

**Table 7:** Computational parameters of different methods.

Method	Model Parameters/MB	FLOPs/GB	Inference Time/s
TCA	\	\	2.76
DAN	1.12	0.006	0.45
EWSNet	1.28	0.007	0.78
DCC	1.87	0.011	0.88
Ref. [35]	1.22	0.006	0.50
MSWCTD (this paper)	1.26	0.007	0.56

## Data Availability

The data that support the findings of this study are available on request.
